# Rapid HIV-1 Disease Progression in Individuals Infected with a Virus Adapted to Its Host Population

**DOI:** 10.1371/journal.pone.0150397

**Published:** 2016-03-08

**Authors:** Jiro Katoh, Ai Kawana-Tachikawa, Akihisa Shimizu, Dayong Zhu, Chungyong Han, Hitomi Nakamura, Michiko Koga, Tadashi Kikuchi, Eisuke Adachi, Tomohiko Koibuchi, George F. Gao, Zabrina L. Brumme, Aikichi Iwamoto

**Affiliations:** 1 Division of Infectious Diseases, Advanced Clinical Research Center, the Institute of Medical Science, the University of Tokyo, Tokyo, Japan; 2 Department of Medical Genome Sciences, Graduate School of Frontier Sciences, the University of Tokyo. Kashiwa-shi, Chiba, Japan; 3 Department of Infectious Disease Control, the International Research Center for Infectious Diseases, the Institute of Medical Science, the University of Tokyo, Tokyo, Japan; 4 Department of Infectious Diseases and Applied Immunology, Hospital, the Institute of Medical Science, the University of Tokyo, Tokyo, Japan; 5 CAS Key Laboratory of Pathogenic Microbiology and Immunology, Institute of Microbiology, Chinese Academy of Sciences, Beijing, China; 6 Faculty of Health Sciences, Simon Fraser University, Burnaby, BC, Canada; 7 British Columbia Centre for Excellence in HIV/AIDS, Vancouver, BC, Canada; 8 Asian Research Center for Infectious Diseases, the Institute of Medical Science, the University of Tokyo, Tokyo, Japan; Helmholtz Zentrum Muenchen—German Research Center for Environmental Health, GERMANY

## Abstract

HIV-1 escape from CTL is predictable based on the Human Leukocyte Antigen (HLA) class I alleles expressed by the host. As such, HIV-1 sequences circulating in a population of hosts will harbor escape mutations specific to the HLA alleles of that population. In theory, this should increase the frequency of escape mutation transmission to persons expressing the restricting HLA allele, thereby compromising host immunity to the incoming HIV-1 strain. However, the clinical impact of infection with HIV-1 containing immune escape mutations has not conclusively been demonstrated. Japan’s population features limited HLA diversity which is driving population-level HIV adaptation: for example, >60% of Japanese express HLA-A*24:02 and its associated Nef-Y135F escape mutation represents the population consensus. As such, Japan is an ideal population in which to examine this phenomenon. Here, we combine genetic and immunological analyses to identify A*24:02-positive individuals likely to have been infected with Y135F-containing HIV-1. Over a ~5 year follow-up, these individuals exhibited significantly lower CD4 counts compared to individuals inferred to have been infected with wild-type HIV-1. Our results support a significant negative clinical impact of pathogen adaptation to host pressures at the population level.

## Introduction

The highly polymorphic human leukocyte antigen class I (HLA-I) molecules present HIV-1-derived peptide epitopes on the surface of infected cells, targeting these for elimination by cytotoxic T lymphocytes (CTL). HLA-Is are thus critical to HIV-1 immune control [1, 2]. They also represent strong evolutionary pressures that drive the selection of CTL escape mutations in the HIV-1 genome [3–6], which act by disrupting intracellular epitope processing [7, 8], abrogating viral peptide-HLA binding [9], or altering interactions between the HLA-bound peptide and the T-cell receptor (TCR)[10]. Critically, CTL escape mutations are highly predictable based on the HLA-I alleles expressed by the host [11, 12]. By extension, HIV-1 sequences circulating in a given host population exhibit adaptations that reflect the HLA-I allele distributions of that population. This in turn means that the frequency of an HLA allele in a population will tend to correlate positively with the frequency of its associated escape mutations in circulation [13], though exceptions apply [14]. Intuitively, higher circulating frequencies of HIV-1 CTL escape mutations should result in their increased transmission to persons expressing the relevant HLA, thereby compromising host cellular immunity to the incoming viral strain. Indeed, observations from high-seroprevalence settings support a role for HLA-driven HIV-1 adaptation in undermining the protective effects of certain HLA-I alleles at the population level [15]. However, the impact of population-level adaptation on HIV-1 disease progression at the individual level has yet to be directly demonstrated.

Japan’s HIV epidemic, largely concentrated in men who have sex with men (MSM), is unique in terms of the limited HLA-I diversity of the host population. For example, >60% of Japanese individuals express HLA-A*24:02 (A*24:02) (http://www.hla.or.jp/). It has been hypothesized that population-level HIV adaptation, and its consequences, may occur more rapidly in populations with limited HLA-I diversity [16]. Indeed, one of the most well-characterized HLA class I-associated escape mutations in HIV, a tyrosine to phenylalanine mutation at Nef codon 135 (Y135F) that is reproducibly selected in A*24:02-expressing individuals [17–22] represents the population consensus sequence in Japan, whereas the global HIV subtype B consensus is the wild-type tyrosine (Y135) (http://www.hiv.lanl.gov/) [17].

Nef codon 135 serves as the N-terminal anchor residue for two overlapping A*24:02-restricted epitopes starting at Nef’s 134^th^ residue: an 8mer (RW8; RYPLTFGW, Nef134-8) [21, 23, 24] and a 10mer (RF10; RYPLTFGWCF; Nef134-10) [17–19, 22, 25]. In theory, CTL responses to these epitopes in A*24:02-expressing persons may differ depending on whether the individual is infected with HIV-1 harboring wild-type (Y135) versus escaped (Y135F) form at this residue: in particular, initial CTL responses to these epitopes in the latter case might be reduced or absent. Furthermore, the presence of Y135F in the infecting HIV-1 strain may signal the presence of other unknown A*24:02-associated escape mutations in other viral epitopes that might also compromise CTL responses to the incoming HIV strain. If this were the case, infection of A*24:02-expressing persons (representing the majority of Japanese individuals) by Y135F-containing HIV-1 (representing the majority of Japanese HIV strains) could bring about graver prognosis than infection by Y135-containing virus. To investigate this, we identified A*24:02-expressing individuals likely to have been infected with escaped (Y135F) versus wild-type (Y135) HIV-1, from within our well-characterized chronic HIV-1 infection cohort, and compared these two groups with respect to their longitudinal HIV-1 clinical outcomes. Our analysis suggested more rapid CD4 decline in A*24:02-expressing individuals inferred to have been infected with Y135F-containing HIV-1.

## Materials and Methods

### Participants

This study comprised 31 A*24:02-positive adults (29 male, 2 female) who were randomly retrospectively selected from participants of a longstanding HIV cohort study based at the Institute of Medical Science at the University of Tokyo (Table 1). All subjects reported unprotected sexual intercourse and were infected with HIV-1 subtype B. Date of infection is not known for study participants. All study patients were untreated with untiretroviral drugs for the duration of followup. In addition to routine viral load and CD4^+^ cell count monitoring, blood samples were separated into peripheral blood mononuclear cell (PBMC) and plasma and preserved in liquid nitrogen or at -80°C, respectively.

**Table 1:** Baseline clinical data of the studied patients.

Patient		First visit			
ID	Gender	Age	Date	CD4*	VL
IMS0367	Male	25	1996-Aug	383	18000
IMS0506	Male	43	2000-Apr	227	140000
IMS0509	Male	34	2000-Jul	590	55000
IMS0525	Female	18	2001-Feb	630	300000
IMS0526	Male	27	2001-Mar	406	9100
IMS0539	Male	30	2001-Aug	376	98000
IMS0541	Male	32	2001-Jul	318	400
IMS0543	Male	32	2001-Aug	442	28000
IMS0573	Male	35	2002-Jul	587	42000
IMS0593	Male	39	2003-Feb	200	2300
IMS0601	Male	35	2003-Apr	397	1200
IMS0617	Male	42	2003-Aug	330	1200
IMS0632	Male	33	2003-Dec	416	4900
IMS0638	Male	17	2004-Mar	770	42000
IMS0649	Male	46	2004-Apr	269	60000
IMS0653	Male	34	2004-Apr	429	4500
IMS0671	Male	21	2004-Aug	319	61000
IMS0692	Male	71	2005-Feb	296	140000
IMS0725	Male	32	2005-Oct	328	220000
IMS0735	Male	29	2005-Dec	620	4400
IMS0738	Male	42	2006-Jan	485	400
IMS0748	Male	51	2006-Mar	329	24000
IMS0758	Male	42	2006-May	218	7400
IMS0765	Male	40	2006-Aug	245	98000
IMS0801	Male	41	2007-Mar	1174	1400
IMS0827	Male	49	2007-Aug	1219	3500
IMS0849	Male	32	2008-Jan	309	1000
IMS0850	Female	40	2008-Jan	401	14000
IMS0871	Male	30	2008-Apr	317	29000
IMS0892	Male	19	2008-Jul	479	970000
IMS0955	Male	48	2009-Feb	341	28000

*CD4 of IMS0525 was measured at 1 week from the first visit.

### Ethics Statement

This study was approved by the internal review board of the Institute of Medical Science at the University of Tokyo (certificates 20-47-210521, 20-31-1120). All study participants were adults and provided written informed consent to participate in the cohort study.

### Plasma HIV-1 RNA sequencing

Plasma viral RNA extraction, primer sets (Nef-1F, Nef-1R, Nef-2F and Nef-2R), RT-PCR amplification and direct sequencing of HIV-1 Nef were performed as previously described [22].

### Proviral DNA pyrosequencing

One microgram of genomic DNA (representing DNA from 1.5 x 10^5^ cells) was subjected to nested-PCR using Platinum *Taq* DNA polymerase High Fidelity (Invitrogen) using first round primers Nef-2F and Nef-2R and second round Nef-specific primers (underlined). The forward primer additionally contained an A-adaptor (italics) and Multiplex Identifier (MID; X_10_): (5’-*CCATCTCATCCCTGCGTGTCTCCGACTCAG*XXXXXXXXXXCTCAGGTACCTTTAAGACCAATG (nt 9028–9050) [26] and the reverse primer contained a B-adaptor (5’-*CCTATCCCCTGTGTGCCTTGGCAGTCTCA*GGAAATGCTAGTTTGCTGTCAAAC, nt 9387–9365). After gel purification, pyrosequencing was performed using the GS-FLX (Titanium kits; Roche Applied Science). GS-FLX sequences were processed by removing primer sequences and extracting sequences corresponding to SF2 nucleotides 9051–9364. Identical reads were collapsed into a single sequence and aligned using MUSCLE [27]. Possible instances of cross-contamination or tag-switching (a known problem during multiplex pyrosequencing [28]) were detected using a genetic distance approach. Nucleotide differences between patient plasma and provirus sequences were identified using MEGA6 [29]. Briefly, if the maximum homology between a patient’s plasma and provirus was <95%, we then examined homology to other patient sequences processed in the same run; if any were >95% homologous these were removed as possible cross-contaminants (four such cases were identified, with putative contaminants occurring at <0.4% in all cases). The remaining sequences were aligned to SF2 nucleotides 9213–9242 (Nef codons 134–143) using a Smith-Waterman algorithm [30, 31] modified to stringently detect insertions/deletions. Insertion/deletion-containing sequences were removed and Y135 frequencies calculated from the resulting alignment.

### HLA typing and haplotype prediction

HLA class I typing was performed by Luminex (WAKFlow HLA Typing kit; Wakunaga Pharmaceutical). HLA-A and -B haplotypes were predicted using the Japanese HLA database (http://www.hla.or.jp/haplo/haplo_search.php?type=haplo&loci=AB&lang=en) using odds-ratios [OR]: if the OR (calculated as [*f*_A1B1_ * *f*_A2B2_] / [*f*_A1B2_ * *f*_A2B1_], where *f*_AiBj_ is the frequency of HLA-A*i* and HLA-B*j*) was > 1, the haplotype was predicted as A1B1 and A2B2; otherwise as A1B2 and A2B1.

### A*24:02 stabilization assay

A TAP2-defective A*24:02-transduced cell line, T2-A24, a kind gift from K. Kuzushima, was cultured in R10 with 0.8 mg/ml G418 (Invitrogen) [32]. Peptide binding to A*24:02 was assessed using a T2-A24 stabilization assay as previously described [17, 22, 32]. B35-14 [NPDIVIYQY] was used as a negative control. In each experiment, the MFI of each peptide was normalized as follows: %max MFI = (MFI_Sample_−MFI_Background_) / (MFI_Max_−MFI_Background_) x 100(%). Three independent experiments were performed.

### Peptides and pHLA tetramers

BirA substrate peptide (BSP)-tagged forms of the A*24:02 heavy chain and β2 microglobulin (β2m) were expressed, refolded with peptides (Nef134-10 [R**Y**PLTFGWCF], Nef134-10(Y135F) [R**F**PLTFGWCF], Nef134-8 [R**Y**PLTFGW], Nef134-8(Y135F) [R**F**PLTFGW]) (Sigma-Genosys) and purified as described [20, 22]. BSP-tagged peptide-HLA complexes (pHLAs) were biotinylated by BirA enzyme (Avidity) and tetramerised by phycoerythrin (PE)- or allophycocyanin (APC)-labelled streptavidin.

### Endogenous antigen presentation assay

CTL clones T26-102, dually specific for Nef134-8(Y135) and Nef134-8(Y135F), and C1-28, dually specific for Nef134-10(Y135) and Nef134-10(Y135F), were previously established from A*24:02-positive HIV-1-infected individuals [22]. CTL clones were stimulated with phytohemagglutinin-L (PHA-L) and maintained with irradiated allogenic PBMCs in R10 with 50U/ml rhIL-2 (R10/50). Their specificity and cross-reactivity were verified by dual-tetramer staining with Tet-8(Y135)-APC and Tet-8(Y135F)-PE for T26-102, or Tet-10(Y135)-APC and Tet-10(Y135F)-PE for C1-28.

Minigene Nef expression vectors pmNef-hRluc-EGFP and pmNef(Y135F)-hRluc-EGFP encoded SF2 Nef codons 123–153 (with or without Y135F, respectively) fused to the N-terminus of *Renilla* Luciferase [22]. Minigene *gag* expression vector pmGag-hRluc-EGFP spanned SF2 p17_Gag_ codons 18–46.

293FT cells, and a derivative line stably expressing A*24:02 (“293FT-A24DRm-CY5”), were cultured in DMEM with 10% FCS. A total of 2 × 10^4^ cells were seeded into 96-well flat-bottom plates and transfected with 100ng of minigene-expression vectors (Fugene HD; Promega) 24 hours later. Transfection efficiency was verified by fluorescence microscopy, and minigene expression was quantified by *Renilla*-Glo Luciferase Assay and Glomax 96 microplate luminometer (Promega) 24 hours later. Transfected cells were co-cultured with 5–7.5 × 10^3^ CTL clones/well for a further 24 hours, after which supernatants were frozen at -80°C for subsequent IFN-γ quantification (Opti-EIA Human IFN-γ ELISA kit; Beckton Dickinson). Three independent experiments were performed, each featuring triplicate co-culture and duplicate ELISA assays per well.

### Peptide stimulation and tetramer staining

Patient PBMCs were thawed and aliquoted into 5 × 10^5^ cells (stimulator) and 1 × 10^6^ cells (responder). 5 × 10^5^ stimulator cells were pulsed with 10 μM Nef134-10 or Nef134-8 peptide for 1 h at 37°C in R10. After extensive washing, stimulator cells were co-cultured with 1 × 10^6^ responder cells and 4 × 10^6^ irradiated (3300 rads) PBMCs from healthy individuals. After 4 days, 50 U/ml rhIL-2 (R&D Systems) was added and cells were cultured for another 10 days in R10/50. Peptide-stimulated PBMCs or CTL clones were stained with 10 μg/ml PE- or APC- labeled tetramers, washed, further stained with anti-CD8-FITC or anti-CD8- PerCP (BD Pharmingen), washed and fixed with 1% paraformaldehyde. Multiparameter flow cytometry was performed using a FACS Calibur (Beckton Dickinson).

### Statistical analysis

Statistical analyses were performed in GraphPad Prism 6 (GraphPad Software). P-values <0.05 were considered statistically significant.

## Results

### T2-A*24:02 stabilization and endogenous antigen presentation assays

**Fig 1:**
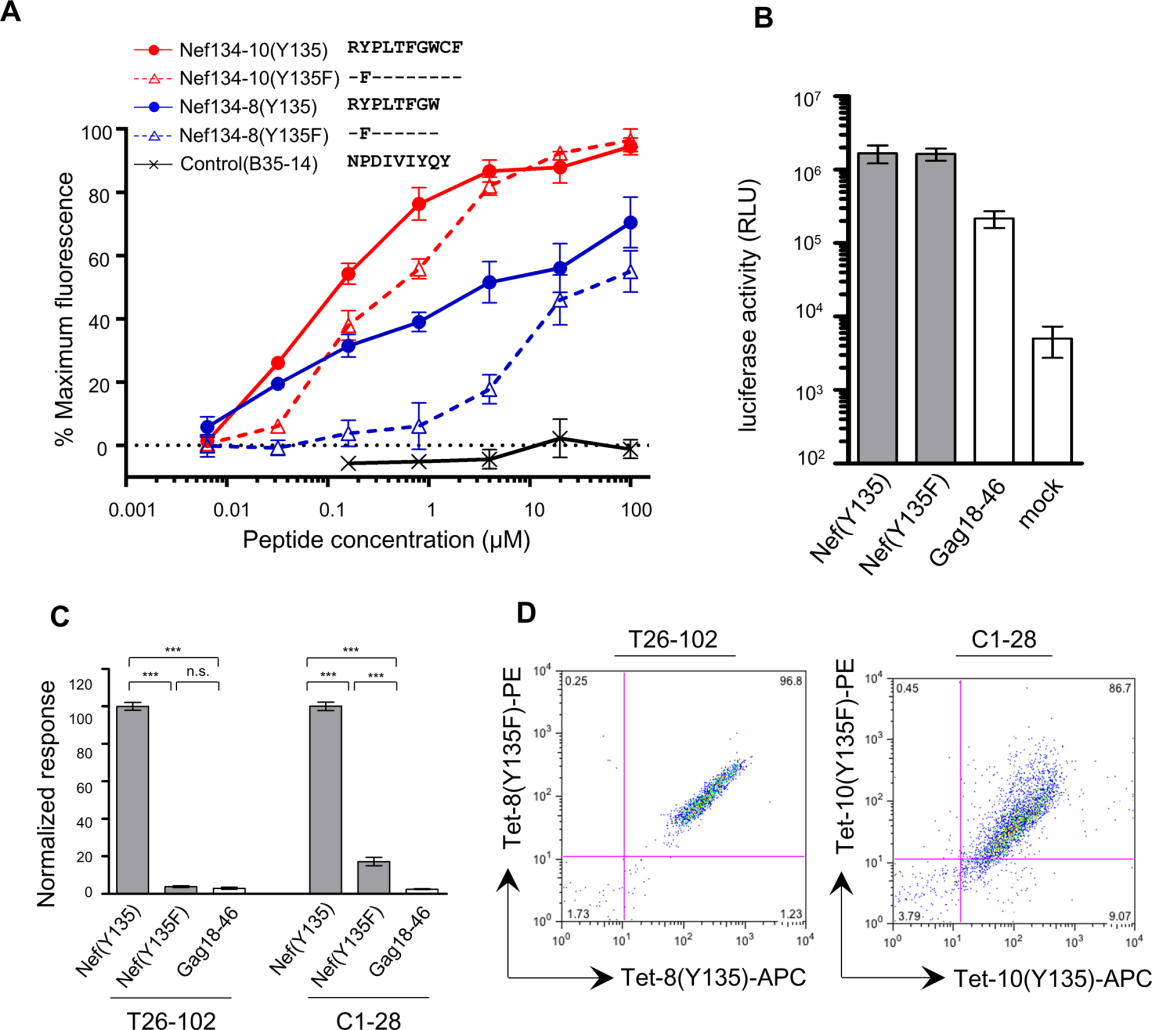
A*24:02 stabilization and endogenous antigen presentation assays. (A) A*24:02 stabilization assay. Binding of Nef134-8 (blue circles) and Nef134-10 (red circles) and their Y135F-containing peptide variants (blue and red triangles, respectively) to T2 cells expressing A*24:02 was measured. Symbols and error bars denote the mean and SEM, respectively. A peptide derived from an HLA-B*35-restricted epitope in HIV-1 reverse transcriptase (B35-14) served as a negative control. % maximum fluorescence was calculated by dividing the MFI of the sample by the maximal MFI in all measured samples in each experiment in each independent experiment. (B) Renilla luciferase (hRluc) activity after transfection of the minigene expressing Nef(Y135), Nef(Y135F), Gag18-46 fused to hRluc or mock controls (culture without transfection). Histograms and error bars denote the mean and SD of triplicate cocultures performed in 3 independent experiments. RLU: Relative light units. (C) Recognition of endogenously expressed epitopes derived from Nef(Y135), Nef(Y135F) and Gag18-46 minigenes by dually specific CTL clones T26-102 or C1-28. Histograms and error bars denote the mean and SEM of replicate IFN-γ ELISA measurements derived from triplicate co-cultures performed in 3 independent experiments. Results are normalized to the mean IFN-γ concentration generated by CTL clones upon co-culture with Nef(Y135)-expressing cells. Asterisks (***) denote p < 0.001 calculated using the Tukey post-test after one-way ANOVA. (D) Double-tetramer staining of CTL clone T26-102 with A*24:02/Nef134-8(Y135) tetramer-allophycocyanin (Tet-8(Y135)-APC) and A*24:02/Nef134-8(Y135F) tetramer-phycoerythrin (Tet-8(Y135F)-PE) (left) and CTL clone C1-28 with A*24:02/Nef134-10(Y135) tetramer-allophycocyanin (Tet-10(Y135)-APC) and A*24:02/Nef134-10(Y135F) tetramer-phycoerythrin (Tet-10(Y135F)-PE) (right).

We first wished to infer how infection with Y135F-containing HIV might affect CTL responses to Nef134-8 and Nef134-10. We began by assessing the ability of these epitopes, with or without Y135F, to stimulate CTL responses in A*24:02-positive individuals. We first evaluated epitope binding to A*24:02 using published methods [17, 22, 32, 33]. Wild-type Nef134-10 exhibited higher binding affinity for A*24:02 than Nef134-8 (Fig 1A). Furthermore, the Y135F mutation reduced binding of the 10mer epitope modestly, but it dramatically reduced binding of the 8mer. This suggests that in individuals infected with Y135F-containing HIV-1, presentation of Nef134-10(Y135F), but not Nef134-8(Y135F), may still occur.

We then evaluated cell surface expression of endogenously-derived epitope using a minigene transfection approach. Luciferase readouts from the fusion constructs indicated that Y135F did not affect minigene transcription and translation (Fig 1B), however this mutation dramatically reduced epitope recognition by CTL clones known to be dually-specific for Y135 and Y135F-containing epitopes (Fig 1C). Specifically, the introduction of Y135F into the minigene abolished the response of CTL clone T26-102, dually specific to Nef134-8(Y135) and Nef134-8(Y135F) (Fig 1C, left), to the background level. Interestingly, weak but significantly higher response than background level (about 20% of the Y135-containing wild type) of CTL clone C1-28, dually specific for Nef134-10(Y135) and Nef134-10(Y135F) (Fig 1C, right), was observed. Together, these results suggest that infection with Y135F-containing HIV-1 may be able to stimulate weak CTL responses to Nef134-10 but not to Nef134-8.

### Plasma HIV-1 genotyping

**Fig 2:**
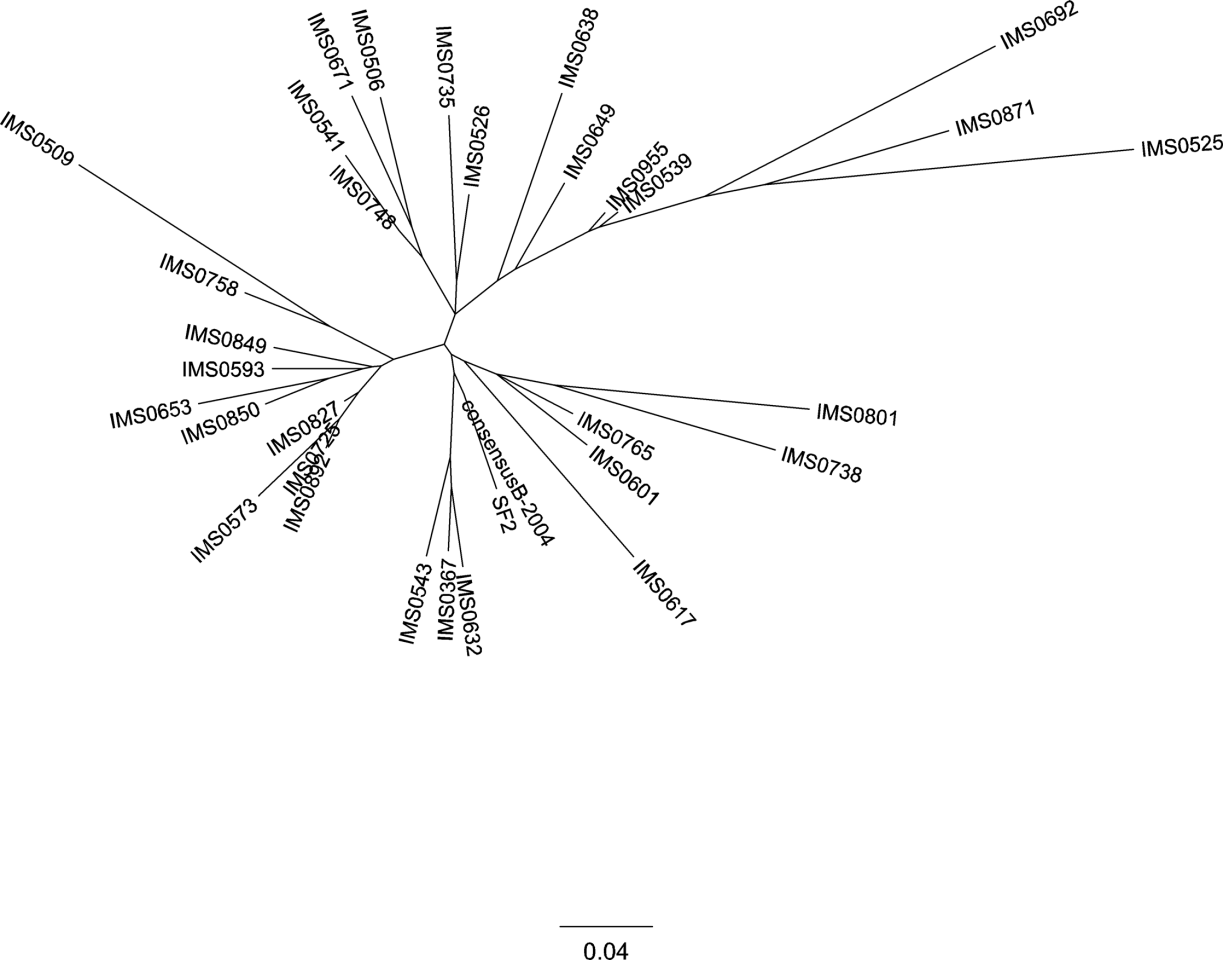
Phylogenetic tree of the *nef* gene nucleotide sequences of HIV-1 in the plasma. HIV-1 *nef* gene in the plasma from the 31 patients in the study was amplified by the RT-PCR and directly sequenced. Published *nef* sequences from SF2 strain (GenBank accession number: K02007) and global subtype B consensus in 2004 (provided at HIV database; http://www.hiv.lanl.gov/content/sequence/NEWALIGN/align.html) were included as controls.

**Fig 3:**
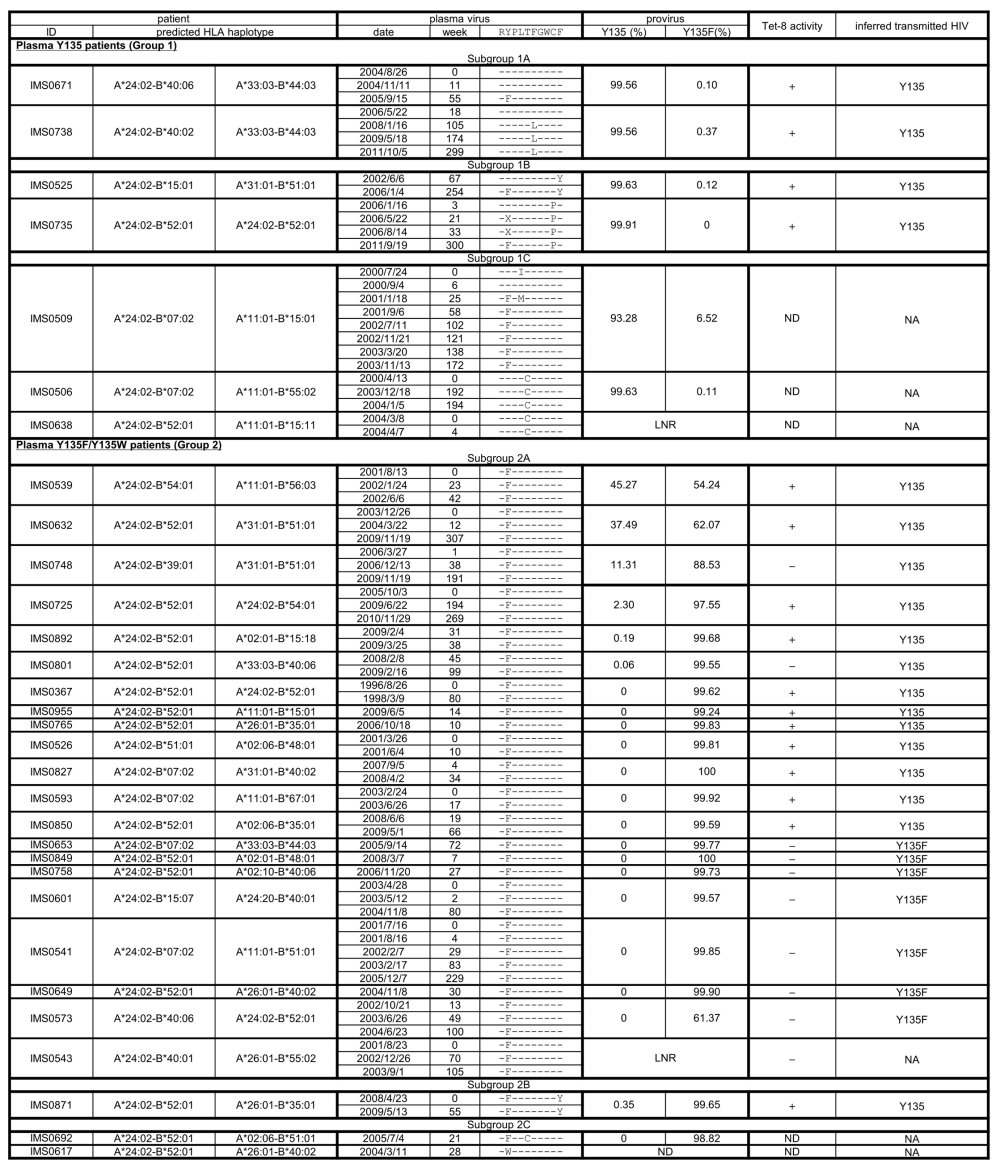
HLA haplotypes, longitudinal plasma viruses and inferred transmitted HIV. Suggested HLA haplotypes and longitudinal plasma viruses of the study patients are shown. Transmitted viruses were inferred based on viral sequence and immune response. LNR, Low Number of Reads; ND, Not Determined; NA: Not applicable.

HIV-1 RNA *nef* sequencing was performed on plasma collected a median of 11 (interquartile range [IQR]: 0–30) weeks from the first visit for all 31 A*24:02-expressing individuals. Patient-derived HIV-1 Nef sequences displayed no extensive phylogenetic clustering (Fig 2), indicating that our study population is unlikely to comprise a large transmission network or otherwise contain large numbers of epidemiologically-linked infections. Median CD4 count and viral loads at sampling were 393 (IQR: 318–590) cells/μl and 1.4 x 10^4^ (IQR: 2.3 x 10^3^–4.2 x 10^4^) copies/ml, respectively, consistent with many participants being in the chronic HIV infection stage at their first (baseline) visit. Nef codon 135 was unambiguously Y135 in 7/31 (23%) individuals (Group 1) or Y135F in 23/31 (74%) and Y135W in 1/31 (3%) (Group 2) (Fig 3). Individuals were further categorized into subgroups A, B and C based on other substitutions within the Nef134-10 epitope at baseline. Subgroups 1A/2A included 23 (74%) individuals who harboured the global wild-type Nef134-10 epitope sequence with or without Y135F (R**Y**PLTFGWCF or R**F**PLTFGWCF). Subgroups 1B/2B included 3 (10%) individuals with substitutions at codons 9 and/or 10 of Nef134-10 (i.e. the two residues downstream of Nef134-8) with or without Y135F. Subgroups 1C/2C included 5 (16%) individuals with substitutions other than Y135F at codon 2 and/or substitutions at central epitope sites.

Follow-up of the four individuals in subgroups 1A and 1B with plasma Y135 at baseline revealed that all subsequently developed escape mutations in this region (Fig 3). The two subgroup 1A individuals subsequently escaped within Nef134-10 (IMS0671 developed Y135F while IMS0738 developed F139L, an escape mutation we identified previously [20]) while the two subgroup 1B patients (IMS0525 and IMS0735) also selected Y135F later in their course. The observation of subsequent escape in these individuals suggest that they may have been recruited into our study at a relatively early infection stage, though it is important to emphasize that infection date is unknown for study participants.

### Sequence-based inference of transmitted HIV-1

Though infection dates (and transmitted HIV-1 sequences) were unknown for study patients, we hypothesized that proviral HIV-1 sequences would contain archived viral sequences, thus yielding clues as to the sequence of the founder virus. Specifically, we hypothesized that the presence of wild-type (Y135) HIV-1 in proviral sequences would indicate that this sequence had previously been present in the patient (i.e. presumably acquired at transmission). Similar approaches have been employed in the context of identifying transmitted drug resistance mutations that may have subsequently reverted in vivo [34–36].

We thus performed Nef proviral deep-sequencing for all patients except IMS0617 (who harbored Y135W) from PBMCs collected a median of 0 (IQR 0–19.5) weeks after the first visit (Fig 3). IMS0543 and IMS0638 were subsequently removed from the analysis due to very low sequence reads. This left 28 patients with a median of 1611 (IQR 1204–2509) reads each. The potential ability of proviral DNA analysis to infer the sequence of the transmitting strain was tested in patient IMS0509 whose initial Y135 plasma viruses were replaced by Y135F variants by week 58: in this patient, Y135-containing proviral sequences could still be detected at 2.75% frequency >12 years later (data not shown).

Consistent with Y135 transmission and subsequent persistence, Y135 provirus dominated (>93%) in all group 1 individuals analyzed (Fig 3). In contrast, 7 of 22 (32%) individuals in group 2 (plasma Y135F) harbored Y135 provirus at frequencies ranging from 0.06–45.27%, identifying these as possible Y135 transmission cases that had subsequently escaped to Y135F *in vivo*. The remaining 15 of 22 (68%) individuals with plasma Y135F harbored no detectable Y135 provirus. This finding suggests possible Y135F transmission (though rapid replacement of Y135 sequences by replicating Y135F virus after selection in the infected host cannot be ruled out).

### Immunological inference of transmitted HIV-1

We next wished to explore whether CTL reactivity to Nef134-8(Y135) and Nef134-10(Y135) might be used to further infer the sequence of the infecting HIV-1 strain within this epitope. To this end, we analysed CD8^+^ T cell responses to Nef134-8(Y135) and Nef134-10(Y135) in patients from subgroups 1A (N = 2), 1B (N = 2), 2A (N = 21) and 2B (N = 1) (the five patients in subgroup 1C/2C were excluded from these and subsequent analyses, as tetramer binding could potentially be affected by non-Y/non-F residues at Nef codon 135 and/or the presence of other substitutions at central epitope positions). We stimulated PBMCs of the 26 patients in groups 1 (N = 4) and 2 (N = 22), sampled a median of 59 [IQR 27.75–106.5] weeks after the first visit, with Nef134-8(Y135) or Nef134-10(Y135) and analysed these by double-tetramer staining (A*24:02/Nef134-8(Y135) [Tet-8] and A*24:02/Nef134-10(Y135) [Tet-10]).

**Fig 4:**
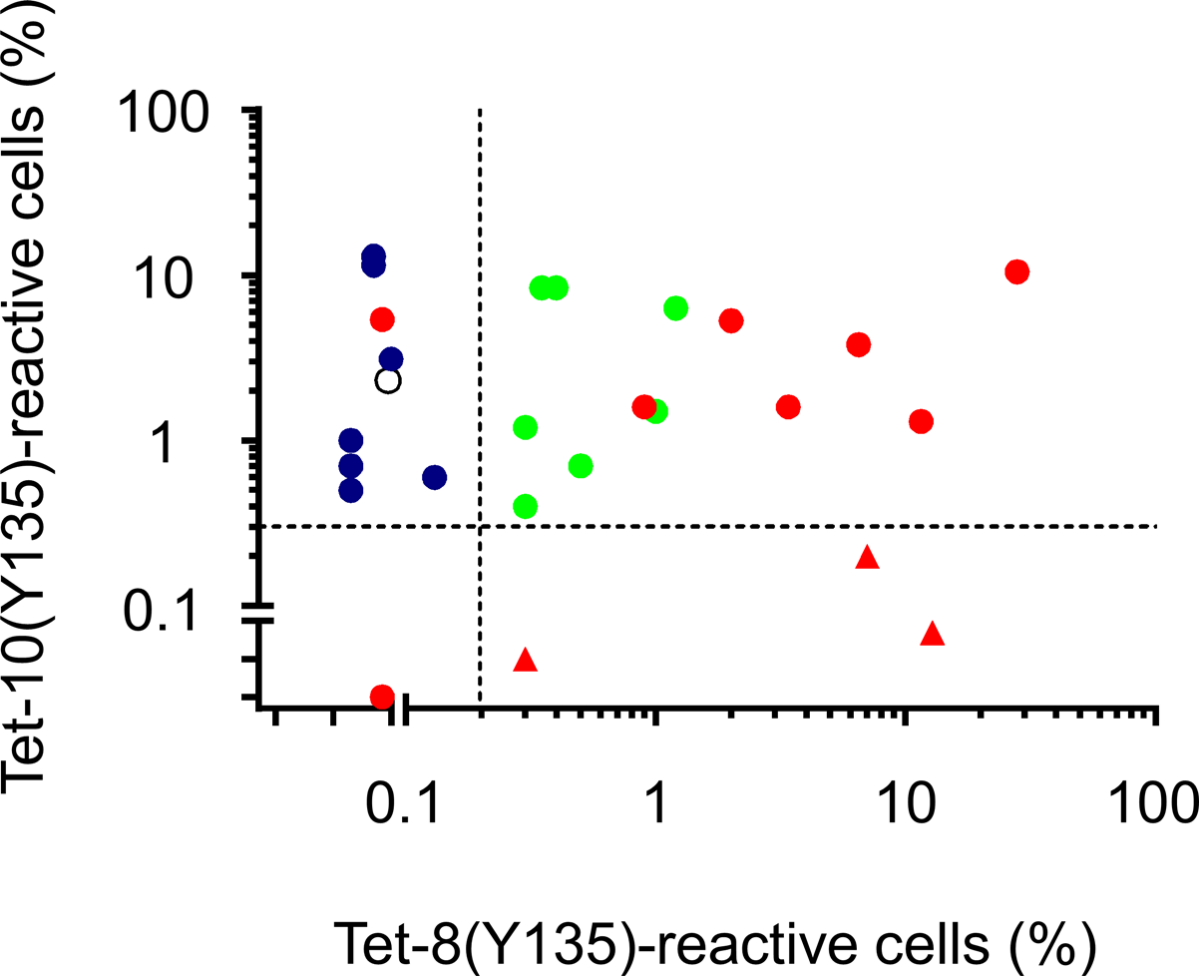
Differential responses to Nef134-8(Y135) and Nef134-10(Y135) in patient groups 1 and 2. % live CD8^+^ cells reactive to Tet-8(Y135) (x-axis) and Tet-10(Y135) (y-axis) after stimulation. Horizontal and vertical dashed lines denote the cut off value for defining tetramer reactivity (mean + 2SD of 5 healthy volunteers). Circles, subgroup 1A and 2A; triangles, subgroup 1B and 2B. Red, Y135-provirus-positive; green, Y135-provirus-negative Tet-8-positive; blue, Y135-provirus-negative Tet-8-negative. Single open circle, Y-135-provirus-LNR, Tet-8-negative.

As expected given their wild-type epitope sequences in plasma HIV-1 RNA, the subgroup 1A patients were both Tet-10- and Tet-8-positive (Figs 3 and 4). Also as expected given the presence of C-terminal mutations within Nef134-10 in plasma HIV-1 RNA (that are beyond the boundaries of the Nef134-8 epitope), the two subgroup 1B patients were Tet-10-negative but Tet-8-positive. Similarly, the only patient within subgroup 2B (IMS0871), who harbored plasma Y135F with a C-terminal mutation in Nef134-10, was also Tet-10-negative and weakly Tet-8-positive. Interestingly, of the 21 subgroup 2A patients who harbored Y135F with no additional substitutions in plasma HIV-1 RNA, 20 (95%) were Tet-10-positive while only 11/21 (52%) were Tet-8-positive (p = 0.0036, Fisher's exact test). Taken together with the observations described above, we inferred that Tet-8-negative responses in this group could reflect two possibilities: either loss of immune memory following Y135-to-Y135F selection *in vivo* (in which case archived Y135 proviruses should be detectable in these patients) or lack of responsiveness to this epitope as a result of infection with HIV-1 containing Y135F.

We then combined the immunological and proviral deep sequence data to infer the transmitted viral sequence. Considering the dramatic negative impact of Y135F on recognition of Nef134-8, we reasoned that the 7 patients who were Y135- provirus-negative and Tet-8-negative represented the most plausible candidates for having been infected with HIV-1 carrying Nef-Y135F. In contrast, the presence of Y135 provirus and/or detectable Nef134-8 CTL responses precludes our ability to rule out infection with wild-type Y135 HIV-1 for the remaining 13 individuals in subgroup 2A.

### Comparison of longitudinal clinical markers in A*24:02-positive patient groups

**Fig 5:**
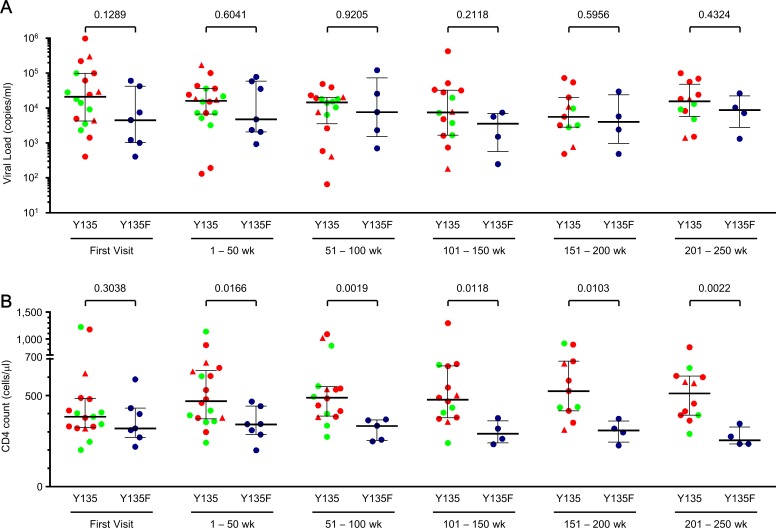
Longitudinal comparison of clinical markers between HLA-A*24:02-positive patients inferred to have acquired the Y135F-containing virus and others. Plasma viral loads (copies/ml; A) and CD4 T cell counts (cells/μl; B) at the first visit and 50 week intervals afterwards are shown for patients for whom de novo infection with HIV-1 containing Y135 could not be ruled out and patients inferred to have acquired Y135F at transmission. Symbols reflect the median of 3 (IQR 2–4) measurements recorded per patient in each period. Symbols and color codes are the same as Fig 4. Horizontal lines and error bars denote the median and IQR for each group. P-values were calculated using the Mann-Whitney test.

Finally, we stratified our A*24:02-positive individuals into those plausibly infected with HIV-1 containing the Nef-Y135F escape mutation (N = 7) and those for whom infection with global wild-type Y135 was probable (or could not be ruled out) (N = 18). Since clinical markers such as plasma viral loads (pVL) and CD4^+^ cell counts fluctuate, we calculated intra-patient median values at 50 week intervals, and analyzed differences between groups over a 250 week period. No significant differences in pVL were observed longitudinally between the two groups (Fig 5A). Notably however, the individuals inferred to have been infected with HIV-1 containing Nef-Y135F exhibited significantly lower CD4^+^ cell counts at all time intervals except the first visit (Fig 5B). Clinical data at all time points are shown in Supporting Information (S1 Fig). Taken together, this suggests that infection with HIV harboring a key A*24:02-restricted Nef escape mutation is associated with significantly poorer long-term CD4 outcomes in A*24:02-expressing individuals.

## Discussion

Japan features low population HLA-I diversity and an HIV-1 epidemic that is highly focused in a single risk group (MSM). The high prevalence (>60%) of A*24:02 in Japan allows the opportunity to examine the impact of population-level viral adaptation to host HLA on HIV progression at the individual level. To this end, we used the A*24:02-associated Nef-Y135F substitution, located at position 2 of the overlapping Nef134-8 and Nef134-10 epitopes, as a marker of viral adaptation to the Japanese population and we integrated immunologic and viral genetic information to identify A*24:02-expressing individuals who likely acquired Y135F-containing HIV-1 at transmission. Based on the major impact of Y135F on Nef134-8 binding and presentation, and the archival nature of proviral HIV sequences, we reasoned that such persons would harbor Y135F in plasma HIV, would have no detectable wild-type Y135 proviruses by deep-sequencing, and would have Nef134-8 tetramer-negative PBMCs. We thus identified 7 individuals likely to have acquired Y135F at transmission and 18 individuals for whom acquisition of wild-type Y135 could not be ruled out (a conservative classification, as the prevalence of Y135F in Japan suggests that it is transmitted in >50% of cases). Notably, the former group exhibited significantly lower CD4 counts compared to the latter over nearly 5 years of follow-up. These observations support the idea that acquisition of HIV-1 “pre-adapted” to one’s cellular immune responses can accelerate disease progression.

Our results also extend our understanding of the Nef-Y135F mutation [21]. Firstly, our assays revealed a stronger negative impact of Y135F on binding and presentation of Nef134-8 compared to Nef134-10. Moreover, patients unable to mount responses to Nef134-10 due to the presence of C-terminal epitope mutations selected Y135F during follow up, presumably as a result of CTL responses against Nef134-8. This suggests that CTL responses to Nef134-8 play a crucial role in selecting the mutation *in vivo*. Furthermore, all 14 patients in whom no wild-type Y135 proviruses could be detected by deep-sequencing were Nef134-10 tetramer-positive, while only 7 of them were Nef134-8 tetramer-positive (p = 0.0058). We showed previously that the tertiary structure of Nef 134–10 bound to A*24:02 is not substantially influenced by the presence of Y135F [20]. Although Nef134-10 presentation is substantially reduced in the presence of Y135F, we speculate that a small amount of Y135F-containing Nef134-10 can still be presented and can still stimulate cognate CTLs. The observation that, in patients with plasma Y135F, TCRs of CD8-positive T cells are often dually-specific for Nef134-10(Y135) and Nef134-10(Y135F) and exhibit a restricted repertoire also supports this notion [19]. The CTL repertoire responding to Nef134-10 thus stimulated may also maintain pressure on Y135F, such that it does not revert to Y135 in A*24:02^+^ persons who acquire it at transmission.

The lack of association between inferred infection with Nef-Y135F and pVL in A*24:02-expressing individuals merits mention, though this is in line with our previous studies indicating no changes in pVL at the population level in Japan over time [37]. Plasma viral loads predict the rate of HIV progression (in terms of CD4 decline) [38] and are thus commonly used as a surrogate of virulence. However, a recent study suggested that population-level HIV-1 adaptation to host HLA may lower the average viral replication capacity [15], which could offset pVL consequences of acquiring escaped HIV. Indeed, we also reported reductions in Gag-protease-mediated viral replication capacity over time among A*24:02-positive patients in our cohort, without discernible effects on viral load [39]. Furthermore, epitope switching from Nef134-10 to the upstream Nef126-10, triggered of Y135F, might also contribute to viral control following escape [22].

CD4 counts were lower for inferred Y135F transmission recipients at all time points except the first visit. Although we cannot formally rule out the possibility that these patients visited the hospital late in their clinical course, a review of their charts did not reveal any information suggestive of late presentation. Furthermore, intra-patient pair-wise genetic distances of group 2 patients were not significantly different between individuals inferred to have been infected with Y135F vs. wild-type Y135 HIV, (data not shown) suggesting that these groups were generally comparable with respect to infection stage. We hypothesize that factors other than viral replication, such as immune activation, could have contributed to the observed accelerated CD4 decline [40].

We interpret our data to suggest that population-level adaptation to the limited HLA diversity in Japan is causing two opposing phenomena. On one hand, these forces may be contributing to reducing viral replication capacity such that modulations in viral load are not apparent at the population level. At the same time, HIV-1 sequences carrying escape mutations may be undermining CTL-mediated viral control, thereby enhancing pathogenesis (in terms of persistently lower CD4 T-cell counts) in persons carrying the most common HLA allele in the population.

The limitations of the study are as follows. First, the sample size was small, which limited statistical power in our study. The modest sample size is attributable to a paucity of retrospective longitudinal specimens and data collected while patients remained antiretroviral-naive (as Japan, like many other global regions, has adopted earlier HIV treatment initiation guidelines), as well as Japan's low HIV incidence and prevalence.Second, infection dates are not known for our cohort patients. As such, we cannot formally exclude the possibility that Y135-provirus- and Tet-8-negative patients were those who had come to the clinic late in their disease. Thirdly, we inferred the founder virus sequence by combining proviral deep-sequencing and immune response data. However, we did not estimate the decay of Y135-provirus and CTL response against Nef134-8(Y135). If these decay rates were random from patient to patient, our inference may have been inappropriate. Lastly, we cannot directly ascribe the observed differences in CD4 count to the Nef-Y135F mutation (though it is intriguing to note that Y135F has been associated with increased HIV-1 replication capacity [41], but also ablation of Nef's HLA class I downregulation function, at least *in vitro* [42]). Rather, we used Y135F as a marker of escaped viruses, and acknowledge that unknown escape mutations could be involved. Further studies in larger cohorts including acute infection cohorts (if available) are needed.

In conclusion, our results suggest that infection with HIV strains harboring escape mutations specific to one’s HLA alleles, transmitted as a result of ongoing HIV adaptation to host populations, may contribute to accelerated clinical progression, presumably because such strains fail to stimulate CTL responses against key epitopes. Results suggest that HIV adaptation may represent challenge to both natural and vaccine-induced immunity in populations with limited HLA diversity and support the importance of prevention, early diagnosis and treatment efforts.

## Supporting Information

S1 Fig All timepoints of clinical markers between HLA-A*24:02-positive patients inferred to have acquired the Y135F-containing virus and others. Symbols and color codes are the same as Figs 4 and 5.
(TIF)
