# Supplementary figures and images for: Rapid HIV-1 Disease Progression in Individuals Infected with a Virus Adapted to Its Host Population
Source: PLoS One. 2016 Mar 8;11(3):e0150397. doi: 10.1371/journal.pone.0150397 (PMC4783116; doi:10.1371/journal.pone.0150397)

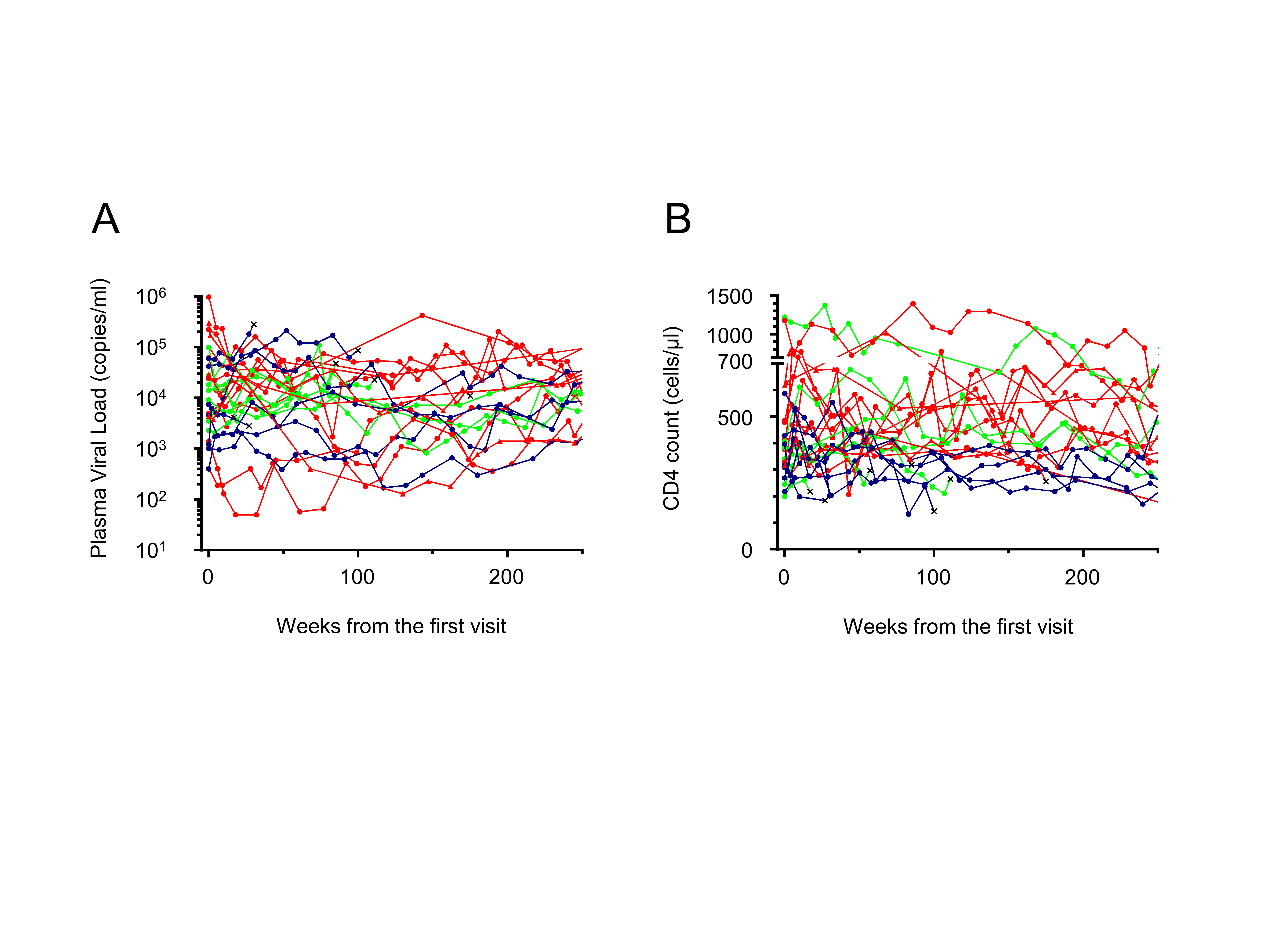

Supplement: S1 Fig — Symbols and color codes are the same as Figs 4 and 5. (TIF) [file pone.0150397.s001.tif]
